# Pro-inflammatory, Th1, Th2, Th17 Cytokines and Dendritic Cells: A Cross-sectional Study in Chronic Periodontitis

**DOI:** 10.1371/journal.pone.0091636

**Published:** 2014-03-26

**Authors:** Giovanna Ribeiro Souto, Celso Martins Queiroz-Junior, Mauro Henrique Nogueira Guimarães de Abreu, Fernando Oliveira Costa, Ricardo Alves Mesquita

**Affiliations:** 1 Department of Oral Surgery and Pathology, School of Dentistry, Universidade Federal de Minas Gerais, Belo Horizonte, Minas Gerais, Brazil; 2 Department of Social and Preventive Dentistry, School of Dentistry, Universidade Federal de Minas Gerais, Belo Horizonte, Minas Gerais, Brazil; University of Leuven, Rega Institute, Belgium

## Abstract

There are a limited number of studies correlating the different stages of dendritic cells (DC) maturation with cytokines in individuals presented chronic periodontitis (CP). The aim of the study was to evaluate the correlation among the expression of IL-2, IL-10, IL-4, IL-6, IFN-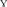, TNF-α, and IL-17A with the presence of DC and mild-moderate or advanced CP. Gingival samples were obtained from 24 individuals with CP and six samples of normal mucosa (NM) overlapping third molar for controls of the levels of cytokines. Periodontal examination was performed. Immunohistochemical staining was carried out, revealing CD1a+ immature, Fator XIIIa+ immature, and CD83+ mature DCs. The inflammatory infiltrate was counted, and the cytokines were measured by flow cytometry. Densities of DCs and inflammatory infiltrate, cytokines, subtypes of CP, and clinical periodontal parameters were correlated and compared. IL-6 expression was correlated positively with the increased numbers of CD1a+ immature DCs. Levels of IL-2, TNF-α, IFN-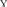, IL-10, and IL-17A were increased when compared with NM. The percentage of sites with clinical attachment level (CAL)>3 were positively correlated with densities of inflammatory infiltrate and negatively correlated with densities of immature DCs. IL-6 can contribute to the increase of the immature DCs in the CP. Higher levels of IL-2, TNF-α, IFN-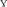, IL-10, and IL-17A cytokines were observed in CP. Higher densities of inflammatory infiltrate as well as lower densities of immature DCs can result in a more severe degree of human CP.

## Introduction

Chronic periodontitis (CP) [1] is the most frequent form of periodontitis [2], [3]. The bacterial biofilm is required, but not sufficient, for disease initiation. Major periodontal tissue destruction results from persistent host inflammatory immune reaction in response to bacteria [4]–[6]. The host inflammatory immune reaction begins when the recognition of the bacterial pathogens occurs by means of antigen-presenting cells, such as dendritic cells (DCs) [7].

Myeloid DCs, also known as conventional DCs, present a strong capability of capturing antigens, which enables them to stimulate T cells [8]. In this context, in CP, DC activation occurs after coming into contact with lipopolysaccharide (LPS) [9], [10] or immune complexes [11] produced by periodontal pathogens [12], [13]. After activation, these DCs become mature, express co-stimulatory molecules, and produce distinct cytokine patterns, such a INF-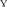 and IL-17, which will determine the selective migration of CD4 T-helper subsets and the subsequent production of characteristic cytokines [7], [11].

CD4 T-cells can be subdivided into Th1, Th2, Th17, and Treg subsets on the basis of their pattern of cytokine production [14], [15]. Protective Th1-related cytokines (Interleukin (IL)-2 and interferon (IFN)-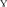) are involved in cellular immune responses [16]-[20]. Th2-related cytokines (IL-4 and IL-10) are associated with humoral immunity and anti-inflammatory properties [16]–[20]. Th2-related cytokinees (IL-4 and IL-10) are associated with humoral immunity and anti-inflammatory properties. However, these arise in a later period of periodontitis and are involved in the chronic progression of the disease[14], [17]–[19]. Th17 was identified as cells that recruited neutrophils and macrophages to participate and amplify the inflammatory reaction [4], [13], [21].

The role of T-helper cells and DCs in the pathogenesis of CP has not yet been fully clarified. There are a limited number of studies correlating the different stages of DC maturation with pro-inflammatory (IL-6 and tumor necrosis factor (TNF)-α), Th1 (IL-2 and interferon (IFN)-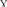), Th2 (IL-4 and IL-10), and Th17 (IL-17A) cytokines in individuals with different stages of CP. In this light, the present study aimed to investigate the correlation among the expression of pro-inflammatory, Th1, Th2, and Th17 cytokines with the presence of DCs in gingival samples of mild-moderate and advanced CP.

## Materials and Methods

### Patients and periodontal samples

This study was approved by the Committee of Ethics in Research from Universidade Federal de Minas Gerais (UFMG), Brazil (423/11). Thirty patients provided written informed consent to participate in this study. All individuals received a full-mouth periodontal examination in which probing depth (PD), clinical attachment level (CAL), bleeding on probing, and oral hygiene index were determined [2], [22]. Individuals presenting four or more teeth with one or more sites containing PD≥4 and CAL≥3 within the same site were diagnosed as CP. Individuals presenting four or more teeth with one or more sites containing CAL≥5 were diagnosed as advanced CP; otherwise, participants were diagnosed as mild-moderate CP [2]. The patients evaluated in this study reported no presence of systemic diseases or immunologic abnormalities. Individuals with smoking habit were excluded [23]. All subjects included in the study had untreated CP at the moment of the sample collection. After diagnostic of mild-moderate or advanced CP, two samples of gingival tissues were collected from each individual during extractions for prosthetic or endodontic reasons in tooth with CP and CAL≥5. The first sample was stored in a buffer (0.4 mM NaCl, 10 mM NaPO4, pH 7.4) containing inhibitors of proteases (0.1 mM PMSF – phenylmethylsulfonyl fluoride – 0.1 mM benzethonium chloride, 10 mM EDTA, and 0.01 mg/mL aprotinin A) and Tween 20 (0.05%), pH 7.4, at a ratio of 1 ml of solution per 100 mg of tissue to perform the cytometric bead array (CBA). The second sample was set in 10% buffered formalin, histologically processed, sectioned, and stained with hematoxylin and eosin or subjected to immunohistochemistry. Due to losses during processing of samples, 24 samples were examined by means of Cytometric Bead Array (CBA), and 22 samples by means of hematoxylin and eosin and immunohistochemistry.

Six samples were collected from the mucosa overlapping the third molar that had been recommended for extraction. These samples were used to control the cytokine levels.

### Detection of tissue cytokines

Multiple gingival tissue cytokines (IL-2, IL-10, IL-4, IL-6, IFN-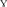, TNF-α, and IL-17A) were simultaneously measured by flow cytometry by means of CBA. The human Th1/Th2/Th17 kit (BD Biosciences, San Jose, CA, USA) was applied following manufacturer instructions. The limit of detection of each cytokine is 0.1 pg/ml (IL-2), 0.03 pg/ml (IL-4), 1.4 pg/ml (IL-6), 0.5 pg/ml (IFN-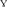), 0.9 pg/ml (TNF-α), 0.8 pg/ml (IL-17A), 16.8 pg/ml (IL-10). Acquisition was performed with a FACSCanto II flow cytometer (BD Biosciences, San Jose, CA, USA). The instrument was checked for sensitivity and overall performance using Cytometer Setup & Tracking beads (BD Biosciences, San Jose, CA, USA) prior to data acquisition. Quantitative results were generated using FCAP Array v1.0.1 software (Soft Flow Inc., Pecs, Hungary).

### Inflammatory assessment

**Figure 1:**
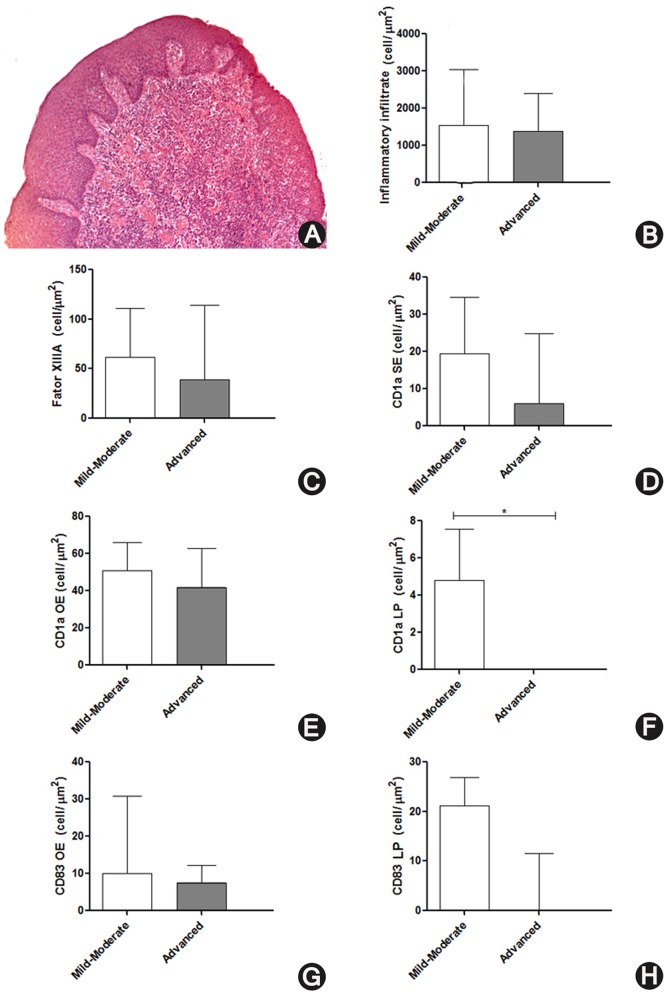
Inflammatory infiltrate cells and dendritic cells (DCs) in individuals with mild-moderate or advanced chronic periodontitis. A) Gingival tissue with intense inflammatory infiltrate on the lamina propria (LP) (haematoxilin-eosin, 50x original magnification). Distribution of the inflammatory infiltrate cells and DCs in groups of individuals with mild-moderate or advanced chronic periodontitis. B) Density of inflammatory infiltrate cells (cells/mm^2^). C) Density of Factor XIIIa+ immature DCs (cells/mm^2^). Density of CD1a+ immature DCs (cells/mm^2^) in the sulcular epithelium (D), oral epithelium (E), and LP (F). Density of CD83+ mature DCs (cells/mm^2^) in the oral epithelium (G) and LP (H). *Mann-Whitney U test, statistically significant difference at P<0.05.

The inflammatory infiltrate in gingival tissue was measured using hematoxylin and eosin stained sections (Figure 1A). These sections were digitized using a microscope (Axio Scoup A1, Zeiss, Göttingen, Germany) at 400x magnification and interfaced to a computer. Cell counts were taken during the sectioning by a blinded examiner (GRS). The mean of inflammatory infiltrate was determined by counting the number of inflammatory cells on the lamina propria (LP) in consecutive fields of all gingival samples (field area: 0.04652 mm^2^) using the Image Tool software (version 3.0, University of Texas Health Science Center, San Antonio, TX). The mean number of cells per unit area (cell number per square millimeters) was obtained. Inflammatory infiltrate density was correlated with periodontal indexes, densities of DCs, and cytokine levels. Furthermore, data were dichotomized by medians: mild and intense inflammatory infiltrate [23]. The periodontal indexes, densities of DCs, and cytokine levels were compared between mild and intense inflammatory infiltrate groups.

### Immunohistochemistry

An immunohistochemical reaction was performed using the streptavidin-biotin standard protocol. Anti-CD1a and anti-Factor XIIIa are considered markers for immature DCs. Anti-CD1a is typical epidermal Langerhans cells (LC) characterized by the expression of Birbeck granules, while Factor XIlla is described as dermal dendritic cells [24]-[26] Anti-CD83 is considered a marker for mature DCs [27]. Serial sections of 3μm from paraffin-embedded blocks were deparaffinized and dehydrated. Antigen retrieval was carried out using the Dewaxing & Antigen Retrieval Buffer 4 (pH 9.0, Spring bioscience, Pleasanton, CA, USA) for 12 minutes at 98°C for anti-CD1a and anti-CD83. Antigen retrieval was carried out using a 10-mM citrate buffer (Laboratory Synth, Diadema, SP, Brazil), pH  = 6.0, for 20 minutes at 98°C for anti-Factor XIIIa. Endogenous peroxidase activity was blocked using 0.3% hydrogen peroxide. Primary antibodies were incubated at room temperature for 1 hour. The following monoclonal antibodies were used: anti-CD1a (clone MTB1; BioSB, Santa Barbara, CA, USA), at a dilution of 1:20; anti-Factor XIIIa (clone AC-1A1; BioSB, Santa Barbara, CA, USA), at a dilution of 1∶500; and anti-CD83 (clone 1H4b; Abcam, Cambridge, UK), at a dilution of 1∶10. Detection was performed using the Advance HRP system (Dako, Carpinteria, CA, EUA) for CD1a, the LSAB system (Dako, Carpinteria, CA, EUA) for Fator XIIIa, and the Reveal system (Spring bioscience, Pleasanton, CA, USA) for CD83. Counter-staining was performed using 3.3′-diaminobenzidine tetrahydrochloride chromogen (DAB, Sigma-Aldrich, St. Louis, MO) and Mayer hematoxylin.

### Immunoexpression analysis and cell counts

Densities of immunolabeled cells (cell number per square millimeters) were calculated for CD1a, Factor XIIIa, and CD83. Positive cell counts were restricted to immunolabeled cells that exhibited well-defined nuclei. The slices were digitized with a microscope (Axio Scoup A1, Zeiss, Göttingen, Germany) at 400x magnification and interfaced to a computer. Cell counts were performed throughout the sections by a blinded examiner (GRS). Areas were delineated using a mouse and measured using the software AxioVision (version 4.8, Zeiss).

### Statistical analyses

SPSS statistic software (SPSS Inc., version 17.0, Chicago, IL, USA) was used for statistical analyses. Normal distribution was tested using the Shapiro-Wilks procedure. In samples with a normal distribution, the Student t test was applied and P values were <0.05. Kruskal–Wallis and Mann-Whitney U tests were used to analyze the samples with abnormal distributions. After Bonferroni correction, the statistical significance was achieved when P values were <0.016. For correlation tests, all samples demonstrated abnormal distribution, and Spearman correlation was applied; the α level was set to 0.05. The correlation was graded according to the Cohen classification as weak (<0.30), moderate (0.30 to 0.50), or strong (>0.50) [28].

Sample calculation considered both type I and II errors. For this, we assumed a 95% confidence interval, 80% power of test, and parameters of values of levels of IL17A obtained in study of periodontal disease of Behfarnia et al. [29].

## Results

### Clinical date

The studied population presented a mean age of 44.50±12.19 years (12 males, mean age: 45.70; and 12 females, mean age: 43.30). Periodontal clinical parameters of the samples are presented in Table 1. In accordance with periodontal parameters for cytokine analysis and DC evaluation, individuals were classified as presenting either mild-moderate CP (n = 10) or advanced CP (n = 14 for cytokines; n = 12 for DCs).

**Table 1:** Periodontal clinical parameters of samples (n = 24).

Clinical Parameters	Median (minimum-maximum)
Number of teeth	16 (4–28)
Number of teeth PD>4 mm	6 (4–21)
Number of teeth CAL>3 mm	12 (4–27)
Number of teeth CAL>5 mm	5 (1–18)
% sites PD>4 mm	19.0 (4.0–100.0)
% sites CAL>3 mm	80.0 (22.0–100.0)
% sites BOP	54.5 (18.0–100.0)

PD - probing depths; CAL - clinical attachment levels; BOP – bleeding on probing.

### Inflammatory infiltrate cells, dendritic cells and CP

**Figure 2:**
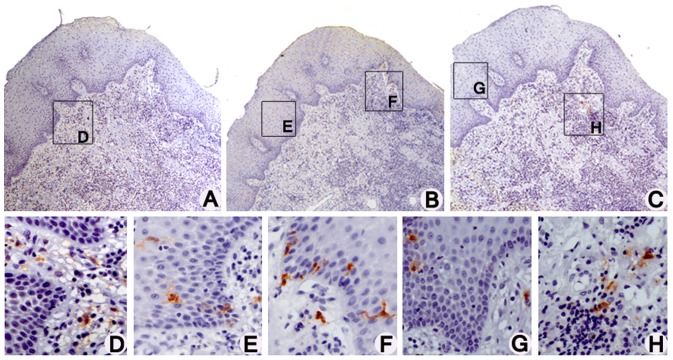
Regions of the gingival tissue evaluated for immature and mature DCs. Immunohistochemical staining of Factor XIIIa+ immature DCs (A), CD1a+ immature DCs (B), and CD83+ mature DCs (C) (streptavidin-biotin, 50x original magnification). Factor XIIIa+ immature DCs could be observed in the lamina propria (LP) region (D). CD1a+ immature DCs could be observed in the oral epithelium (E), sulcular epithelium and LP regions (F). CD83+ mature DCs could be observed in the oral epithelium (G) and LP regions (H) (streptavidin-biotin, 400x original magnification).

All regions of the gingival tissue were evaluated for each antibody. Factor XIIIa+ immature DCs could be observed only in the LP (Figure 2A and 2D); CD1a+ immature DCs could only be observed in the oral epithelium (OE), sulcular epithelium (SE), and LP (Figure. 2B, 2E and 2F); while CD83+ mature DCs could be observed in the OE and LP regions (Figure 2C, 2G, 2H). CD1a+ immature DCs showed cytoplasmic processes in the OE and SE (Figure 2E and 2F, respectively), CD1a+ immature DCs in the LP (Figure 2F), Factor XIIIa+ immature DCs in the LP (Figure 2D), and CD83+ mature DCs in the OE and LP (Figure 2G and 2H, respectively), all of which presented a rounded or oval shape. The density of inflammatory cells and DCs was compared between mild-moderate CP and advanced CP (Figure 1B, 1C, 1D, 1E, 1F, 1G, and 1H). Despite the lack of statistical significance, the group of individuals with advanced CP presented slightly less inflammatory infiltrate cells and DCs than did individuals with mild-moderate CP. A statistically significant difference could be observed for the CD1a+ immature DCs of the LP (Figure 1F).

Regarding possible altered phenotypes between the female and male genders, when comparing the density of inflammatory infiltrate cells and the density of immature and mature DCs, no significant differences between genders could be observed.

### Intensity of inflammatory infiltrate and DC densities

The present study evaluated whether or not the density of inflammatory infiltrate cells influenced the quantity of DCs. Eleven samples were grouped in mild inflammatory infiltrate and eleven in intense inflammatory infiltrate. The density of Factor XIIIa+ immature DCs was higher in individuals presenting an intense, as compared to mild, inflammatory infiltrate density (P<0.05) (table 2). For this reason, correlations between inflammatory infiltrate cells and DCs also were performed. In this regard, there was a positive correlation (P<0.05) between the density of Factor XIIIa+ immature DCs and inflammatory infiltrate cells. Furthermore, positive correlations (P<0.05) could be observed between the density of CD1a+ immature DCs in the OE and CD1a+ immature DCs in the LP with inflammatory infiltrate cells. By contrast, negative correlations (P<0.05) were observed between the density of CD83+ mature DCs in the OE and inflammatory infiltrate cells.

**Table 2:** Densities of dendritic cells (DCs) in gingival samples of individuals presenting mild and intense inflammatory infiltrate.

DC (cells/mm^2^)	mild inflammatory infiltrate (n = 11)	intense inflammatory infiltrate (n = 11)
Fator XIIIA*	38.64 (± 23.84)	99.60 (± 63.81)
CD1a SE	15.47 (± 14.88)	18.91 (± 23.42)
CD1a OE	50.91 (± 28.21)	48.87 (± 22.05)
CD1a LP	1.81 (± 2.62)	5.52 (± 7.08)
CD83 OE	11.18 (± 13.86)	17.02 (± 15.95)
CD83 LP	15.40 (± 21.72)	7.67 (± 11.11)

SE – sulcular epithelium; OE – oral epithelium; LP – lamina própria.

* p<0.05, Student t test.

### Pro-inflammatory, Th1, Th2, and Th17 cytokine profiles correlated with CP

**Figure 3:**
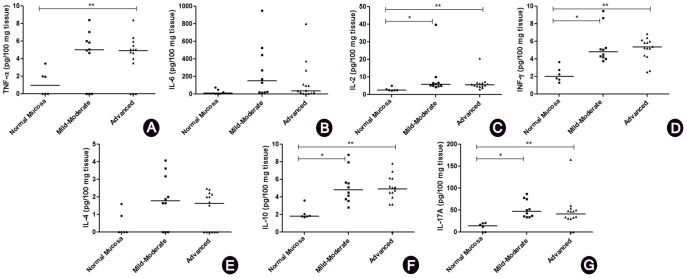
Cytokine levels in individuals with mild-moderate or advanced chronic periodontitis and normal mucosa. Levels of TNF-α (A), IL-6 (B), IL-2 (C), INF-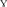 (D), IL-4 (E), IL-10 (F), and IL-17A (G). *Statistically significant difference at P<0.016 in normal mucosa *versus* mild-moderate chronic periodontitis. **Statistically significant difference at P<0.016, in normal mucosa *versus* advanced chronic periodontitis.

Pro-inflammatory (IL-6 and TNF-α), Th1 (IL-2 and INF-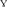), Th2 (IL-4 and IL-10), and Th17 (IL-17A) cytokine profiles were compared among the groups with mild-moderate CP (n = 10), advanced CP (n = 14), and NM (n = 6) (Figure 3). IL-2, INF-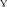, IL-10, and IL-17A levels were higher (P<0.016) in the individuals presenting mild-moderate and advanced CP, when compared to NM (Figure 3C, 3D, 3F and 3G, respectively). By contrast, TNF-α levels were higher (p<0.016) in the individuals presenting advanced CP, as compared to NM (Figure 3A). In addition, cytokines correlations was performed and presented in the table 3. Pro-inflammatory, Th1, Th2, and Th17 profiles of cytokines were correlated among themselves. Then, the samples were grouped according to the classification of CP and cytokines were correlated (table 3). No differences between females and males could be observed when comparing cytokines.

**Table 3:** Correlations between pro-inflammatory, Th1, Th2 and Th7 profiles of cytokines in all individuals with chronic periodontitis (CP), or in individuals with mild-moderate CP and individuals with advanced CP.

	Pro-inflammatory cytokines		Th1 cytokines		Th2 cytokines		Th17 cytokines
**Individuals with CP (n = 24)**	TNF-α	IL-6	IFN- γ	IL-2	IL-4	IL-10	IL-17
TNF-α	1	–0.20	0.42*	0.65*	0.75*	0.70*	0.60*
IL-6		1	0.07	–0.09	0.03	–0.03	0.04
IFN-γ			1	0.59*	0.59*	0.56*	0.50*
IL-2				1	0.58*	0.73*	0.52*
IL-4					1	0.55*	0.63*
IL-10						1	0.50*
**Mild-moderate CP group (n = 10)**	TNF-α	IL-6	IFN-γ	IL-2	IL-4	IL-10	IL-17
TNF-α	1	–0.05	0.47	0.76*	0.61	0.86*	0.65*
IL-6		1	0.51	0.00	0.23	0.20	0.34
IFN- 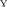			1	0.43	0.78*	0.67*	0.72*
IL-2				1	0.51	0.75*	0.75*
IL-4					1	0.61	0.58
IL-10						1	0.87*
**Advanced CP group (n = 14)**	TNF-α	IL-6	IFN-γ	IL-2	IL-4	IL-10	IL-17
TNF-α	1	–0.24	0.27	0.52	0.86*	0.54*	0.64*
IL-6		1	–0.17	–0.11	–0.16	–0.21	10.15
IFN- 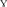			1	0.65*	0.37	0.38	0.44
IL-2				1	0.55*	0.60*	0.43
IL-4					1	0.42	0.66*
IL-10						1	0.40

* p<0.05, Spearman correlation coefficient.

### Correlations among cytokine levels, dendritic cells, inflammatory infiltrate cells, and clinical periodontal parameters of CP

Positive correlation (P<0.05) could be observed between IL-6 and the density of CD1a+ immature DCs, as well as the density of inflammatory infiltrate cells, in both the SE and LP. A positive correlation (P<0.05) could also be observed between IL-6 and the percentage of sites with PD>4. By contrast, a negative correlation (P<0.05) could be identified between IL-2 and the percentage of sites with CAL>3.

Positive correlations (p<0.05) could be observed between the percentage of sites with PD>4 and the density of inflammatory infiltrate cells, CD1a+ immature DCs in the SE, and Factor XIIIa+ immature DCs. Moreover, a positive correlation (p<0.05) could also be identified between the percentage of sites with CAL>3 and the density of inflammatory infiltrate cells. However, negative correlations (p<0.05) could be observed between the number of teeth with CAL>3 and CD1a+ immature DCs in the SE, as well as between the number of teeth with CAL>5 and CD1a+ immature DCs in the SE and LP.

## Discussion

DCs are the most potent antigen-presenting cells (APCs), whose function is to initiate an adaptive immune response. Activation and maturation of DCs are induced by infectious agents and inflammatory products [7], [30], as well as by host cytokines [31], [32]. Cytokines affect DC differentiation and are important in controlling states of perpetual inflammation, as can be seen in CP, when DCs are continuously exposed to antigens [33]. Prior reports have shown that, in regions such as the lymph nodes, spleen, and liver, where DCs are continually exposed to potential antigens, IL-6 avoids a state of perpetual inflammation and protects central immune organs from overstimulation [34], [35]. In this regard, *in vitro* studies have shown that IL-6 plays a major role in maintaining immature DCs and in blocking DC maturation. IL-6-mediated negative feedback may well contribute to the down-regulation of the immune response initiated by pathogens or in persistent infections [36]. In fact, in the present study, the IL-6 expression in gingival tissues was positively correlated with the increased number of immature DCs in patients presenting CP. It is therefore possible to conclude that IL-6 may well contribute to the inhibition of DC maturation in human CP.

In addition, it is known that macrophages are important APCs. It was demonstrate that these cells can contribute to innate immunity employing various cytokines and chemokines [13], [37]. However, it was not possible to evaluate macrophages in the present study. It was demonstrated that responses of the macrophages and DCs in the production of cytokines and chemokines were similar for the various microbial challenges [37]. Huang et al. [37] demonstrated that only response of immature DC to polymicrobial challenge could contribute to production of IL-6 [37]. Furthermore, others studies are necessary to understand mechanisms that affect the relation between immature DCs and IL-6.

IL-6 levels showed no increase when compared with NM, whereas the levels of IL-2, TNF-α, INF-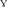, IL-10, and IL-17A did present a significant increase. It is well-known that, when there is an absence of cytokines, such as TNF-α, IL-12, or IFN-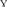, the IL-6 cytokine can favor immune tolerance, in turn contributing to homeostasis [34]. By contrast, as can be witnessed with other pro-inflammatory cytokines and in inflammatory conditions, IL-6 can aggravate the inflammatory disease pattern [34]. Therefore, it can be proposed that the increased expression of IL-2, TNF-α, INF-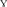, IL-10, and IL-17A cytokines can influence the role of IL-6, in turn causing alterations in host immune responses, thus contributing to the pathogenesis of CP.

In line with these results, the IL-6 expression proved to be positively correlated with PD. Guillot et al. [38] and Baker, et al [39] reported on the role of IL-6 in the resorption of the alveolar bone in periodontal disease. IL-6 also induces bone resorption, both alone and together with other bone-resorption agents [40]. However, Moutsopoulos et al., [10] demonstrated that only IL-17 was positively correlated with bone loss in CP, suggesting that the Th17 subset may well drive or contribute to bone destruction. It could also be observed that female mice without an IL-17 receptor proved to be much more susceptible to bone loss in periodontal disease than in males, demonstrating a gender-dependent effect of IL-17 signaling [41]. In addition, estrogen loss resulted in an IL-6-mediated stimulation of osteoclastogenesis, which suggests a mechanism for the increased bone resorption in postmenopausal osteoporosis [42]. Nevertheless, although the present study’s results demonstrated that IL-17A, IL-2, TNF-α, INF-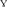, and IL-10 levels were higher in CP when compared to NM, it was impossible to demonstrate any form of altered phenotypes between genders when comparing cytokine levels, the density of inflammatory infiltrate cells, or the densities of immature and mature DCs.

Th1-related cytokines are related to the early process of periodontal disease [19], [43], while Th2-related cytokines were related to the later stages of the periodontal disease [14], [19]. In addition, Th17 cells amplified the inflammatory destruction through the recruitment of inflammatory cells to the target site [4], [13], [21]. In the present study, IL-2 was negatively related to CAL, which is related to advanced CP, resulting in a greater severity of the disease [2]. Accordingly, our data showed that, in individuals presenting mild-moderate CP, the Th17 cytokine (IL-17) proved to be positively correlated with the Th1 (IL-2 and INF-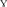) cytokine profile, while in individuals presenting advanced CP these correlations were not observed. This result suggests that the balance of Th17/Th1 cytokines is involved in the amplification of inflammatory reaction, especially in the early stages of periodontal disease.

Differences in cytokines levels can be observed in accordance with the applied methodology. Analysis of the IL-6 levels in gingival crevicular fluid (GCF) of patients with CP showed a decrease in disease sites, as compared to non-diseased sites [37], [44]. However, an increase in IL-6 levels could be identified in the gingival connective tissue adjacent to the intra-bony pocket [38] and the GCF of sites with CP [45]–[47]. The present study’s data showed no significant differences in IL-4 and IL-6 but did register a significant increase in TNF-α, IL-2, INF-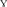, IL-10, and IL-17 levels in tissues with CP when compared to the NM. By contrast, IL-4 [29] and IFN-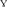 [29], [47] levels showed a significant reduction in patients with CP, as compared to those of healthy gingival samples, while other studies demonstrated an increase in IL-4 [4] and IL-6 [44] after treatment, suggesting a protective role for these cytokines.

In most studies evaluating DCs in periodontal disease, an increase in these cells can be observed in diseased tissues, as compared to healthy samples, although a decrease in the later processes of the disease can also be seen [48]–[55]. In the present study, positive correlations could be identified between immature DCs and probing depth. Likewise, fewer immature DCs could be found in the group of individuals with advanced CP. Although no comparisons were performed between diseased tissue and NM, these data indicate that increased numbers of immature DCs are associated with the initial stage of periodontal disease. In addition, negative correlations could be observed between immature DCs and CAL>3, as well as between immature DCs and CAL>5, thus suggesting that these cells decrease with the severity of CP.

The present study found an increase in the percentage of sites with PD>4 and CAL >3, and IL-6 levels and immature DCs with higher inflammatory infiltrate. In a previous study by our research group, an increase in immature DCs with inflammatory infiltrate was identified, while mature DCs proved to be positively correlated with mild inflammatory infiltrate in samples presenting chronic gingivitis [23]. In this study, a decrease in mature DCs, with higher inflammatory infiltrate, could be observed. This observation was also associated with the absence of these cells in the SE, a region characterized by intense inflammatory infiltrate in the adjacent LP. Therefore, the inverse relation of mature DCs with inflammatory infiltrate may well explain their absence in the SE.

In conclusion, the IL-6 can contribute to the increase of the immature DCs in the CP, in turn impacting the inflammatory response. Higher levels of IL-2, TNF-α, INF-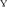, IL-10, and IL-17A cytokines in the gingival tissue are related to human CP, while higher densities of inflammatory infiltrate and lower densities of immature and mature DCs may well result in much more severe degree of human CP.
